# The Obese Brain Athlete: Self-Regulation of the Anterior Insula in Adiposity

**DOI:** 10.1371/journal.pone.0042570

**Published:** 2012-08-08

**Authors:** Sabine Frank, Sangkyun Lee, Hubert Preissl, Bernd Schultes, Niels Birbaumer, Ralf Veit

**Affiliations:** 1 MEG Center, University of Tübingen, Tübingen, Germany; 2 Institute of Medical Psychology and Behavioral Neurobiology, University of Tübingen, Tübingen, Germany; 3 Department of Obstetrics and Gynecology, University of Arkansas for Medical Sciences, Little Rock, Arkansas, United States of America; 4 Interdisciplinary Obesity Center, Cantonal Hospital St. Gallen, CH-9400 Rorschach, Switzerland; 5 Ospedale San Camillo, Istituto di Ricovero e Cura a Carattere Scientifico, Venezia-Lido, Italy; University of Cordoba, Spain

## Abstract

The anterior insular cortex (AIC) is involved in emotional processes and gustatory functions which can be examined by imaging techniques. Such imaging studies showed increased activation in the insula in response to food stimuli as well as a differential activation in lean and obese people. Additionally, studies investigating lean subjects established the voluntary regulation of the insula by a real-time functional magnetic resonance imaging-brain computer interface (rtfMRI-BCI) approach. In this exploratory study, 11 lean and 10 obese healthy, male participants were investigated in a rtfMRI-BCI protocol. During the training sessions, all obese participants were able to regulate the activity of the AIC voluntarily, while four lean participants were not able to regulate at all. In successful regulators, functional connectivity during regulation vs. relaxation between the AIC and all other regions of the brain was determined by a seed voxel approach. Lean in comparison to obese regulators showed stronger connectivity in cingular and temporal cortices during regulation. We conclude, that obese people possess an improved capacity to self-regulate the anterior insula, a brain system tightly related to bodily awareness and gustatory functions.

## Introduction

The number of obese subjects has more than doubled since 1980 and this development is not only a problem of industrialized countries anymore [1]. As the fifth leading risk factor of mortality, obesity is, according to the world health organization, associated with at least 2.8 million deaths each year, resulting from increased risk for illnesses like type 2 diabetes, coronary heart disease or different kinds of cancer [2]. Until now, treatment options for weight loss and especially long-lasting weight maintenance are limited. A closer understanding of brain functions related to eating behavior may help to develop more effective therapies for obesity. Brain networks related to homeostatic, reward and gustatory processes regulating eating behavior are of particular interest. The anterior insular cortex (AIC) is a brain region that is involved in various functions including gustatory perception [3] and the processing of food items [4], [5]. Among other brain regions involved in reward processing, the anterior part of the insular cortex shows enhanced activation in overweight compared to lean subjects in response to hedonic food stimuli [6]–[9]. Moreover, it has been shown that after successful weight loss interventions the AIC and the neighboring frontal operculum exhibit altered activation elicited by food cues [10]. But not only cue-related data show BMI-dependent differences. Resting state functional connectivity in the insular cortex, which is a part of the temporal lobe network, is decreased in obese subjects [11].

Based on these neuronal differences found between obese and lean subjects in brain functions, it is intriguing to speculate about possible brain related intervention strategies for weight loss and for long-lasting weight maintenance. One approach can be based on the direct regulation of brain activity with neurofeedback training using the technology of fMRI based Brain Computer Interface (BCI) [12], [13]. BCI approaches based on real-time functional magnetic resonance imaging (rtfMRI) allow to voluntarily control localized brain regions including subcortical structures via BCI-training and are already applied in therapeutic interventions for chronic pain, stroke, schizophrenia or seizure disorder [14], [15]. Based on these findings and the stronger responsiveness of the insular cortex to food cues in obese, we assume that this region is more likely to be activated in obese subjects. Therefore, we chose the AIC bilaterally as target for a rtfMRI training. We hypothesized that obese people show increased insula regulation, since obese persons are more likely to focus on hedonic properties and show a particular responsiveness when stimulated with hedonic food specific stimuli. We therefore expected a general easier excitability of the AIC what presumably also influence the functional connectivity of brain areas connected to the AIC.

## Methods

### Participants

Twenty one male, healthy participants (11 lean, 10 obese) were included in this study (Table 1). The two groups were age matched. None of the participants suffered from any psychiatric or neurological disease or was under current medication.

**Table 1:** Participants characteristics.

	Lean	Obese	p-value
**N**	11	10	
**BMI**	23.90±0.509	34.49±0.909	<0.001
**Age**	26.27±0.702	26.20±0.998	0.952

BMI = Body Mass Index.

### Ethic Statement

All participants gave written informed consent and the study protocol was approved by the ethics committee of the Medical Faculty of the University of Tübingen.

### Study Design

Participants underwent a rtfMRI-BCI training on two consecutive days. The training took place in the morning between 9 and 11 am after an overnight fast of at least 12 hours. All participants were given a standardized meal 30 minutes before the rtfMRI training (FRESUBIN® protein energy Drink, multifruit, 300 kcal, 200 ml).

### Imaging Procedures

During the 3 training sessions per day (7 minutes each session, 2 days and 6 sessions in whole) participants were asked to increase the activity of the AIC (upregulation) alternated with a relaxation (no regulation) phase. For the upregulation period participants were instructed to think about something subjectively emotional either positive or negative. We emphasized that any strategy to affect the feedback signal in the correct upward direction should be used. In the relaxation phase participants were supposed to count backward starting from ten. One session consisted of 7 blocks of 30 seconds of upregulation, followed by 30 seconds of relaxation. The left and right AIC were defined anatomically to delineate the region of interest (ROI) for the feedback signal. Additionally, a reference ROI not involved in emotional or visual processing (including mainly motor areas) was chosen in order to control for non-specific effects and any unspecific activation. The feedback signal was computed from the time series of the ROIs and was calculated using the following equation:

(AIC_left_(BOLD_reg_−BOLD_relax_)+AIC_right_(BOLD_reg_−BOLD_relax_))/2−ROI_ref_(BOLD_reg_−BOLD_relax_), ROI_ref_ representing the respective BOLD-signal in the reference area, BOLD_reg_ the BOLD-signal during the upregulation phase, and BOLD_relax_ the BOLD signal during the relaxation phase. In the scanner, the participants saw a thermometer bar with a blue or red background indicating the task. During the red background participants were instructed to upregulate, i.e. increase the activity, and the feedback was provided in real time as a moving bar indicating the current activity. During the relaxation phase (blue background) the bar did not move to avoid distraction. For the signal analysis in real time, including online movement correction, the Turbo-BrainVoyager software (Brain Innovations, Maastricht, The Netherlands) was used. A custom-made visualization software was implemented to provide the visual feedback. For detailed information see Sitaram et al. [16].

Data were obtained using a 3T scanner (Tim Trio, Siemens, Erlangen, Germany). FMRI imaging of the whole brain was acquired by echo planar imaging using the following parameters: TR = 1.5s, TE = 30 ms, flip angle = 70°, 16 slices, FOV = 210, image matrix = 64×64, voxel size 3×3×5 mm^3^. Each session lasted 290 scans. On both days a high resolution structural image was acquired.

### Offline Imaging Analysis

Standard preprocessing including realignment, coregistration to the anatomical T1 weighted image, normalization into Montreal Neurological Institute (MNI) space (3×3×3 mm^3^), and Gaussian spatial smoothing (FWHM: 12 mm) was performed using SPM8 (http://www.fil.ion.ucl.ac.uk/spm/). Data were high-pass filtered (cut off: 128 s) and auto correlation corrected (AR(1)). For each condition a separate regressor was modeled using a canonical hemodynamic response function. The contrast upregulation – relaxation was calculated for each subject and the resulting contrast images were entered into a second level GLM analysis to examine the effect of the regulation in the whole brain. For these analyses, effects were considered as significant using a threshold of *P*_FWE_<0.05 (family wise error corrected).

To calculate the regulation ability index (RAI), the time series of the three ROIs (left and right AIC and reference region) were extracted from the Turbo-BrainVoyager and further analyzed using SPSS 18 (SPSS Inc, Chicago, IL). First, the percent signal change during the upregulation period in proportion to the relaxation period was calculated for each session and ROI. After that, the regulation ability for each session was calculated based on the following function: (AIC_left(%change)_+AIC_right(%change)_/2)-ROI_ref(%change)_. These data were then averaged across all sessions to create the RAI over all sessions.

### Functional Connectivity

For the functional connectivity analysis the left and right AIC were used as seeds. Data, previously preprocessed and modeled in a first level analysis using SPM, were further analyzed using the *conn* toolbox (http://web.mit.edu/swg/software.htm). The first level analysis defined the functional connectivity of the different seeds (left and right AIC) and conditions (upregulation vs. relaxation) for each participant corrected for possible confounds (CSF and realignment parameters). The second level analysis included the different groups (lean vs. obese regulators), conditions and seeds. GLMs were calculated using the contrast images reflecting the connectivity change of the upregulation vs. the relaxation period. Results were considered significant using *P*<0.001, uncorrected.

### Behavioral Data

Subjective hunger was measured on a 100 mm visual analogue scale (VAS) at both scanning days before the measurements started. The German version of the Three Factor Eating Questionnaire (TFEQ) was used to examine the extent of the trait variables *restraint eating*, *disinhibition* (tendency to overeat) and *experienced hunger* [17]. Additionally, actual (state) and general (trait) food craving were assessed with the Food Craving Questionnaire (FCQ) [18]. Two sample T-tests were performed using SPSS to examine group differences with a significance threshold of *P*<0.05. Additionally, mood was examined by subjective ratings of anxiety, power of concentration, anger, sadness, happiness, nervousness, and weakness on a 100 mm VAS. Multivariate ANOVA were calculated to examine group differences to the four time points.

## Results

### Regulation Ability

**Figure 1:**
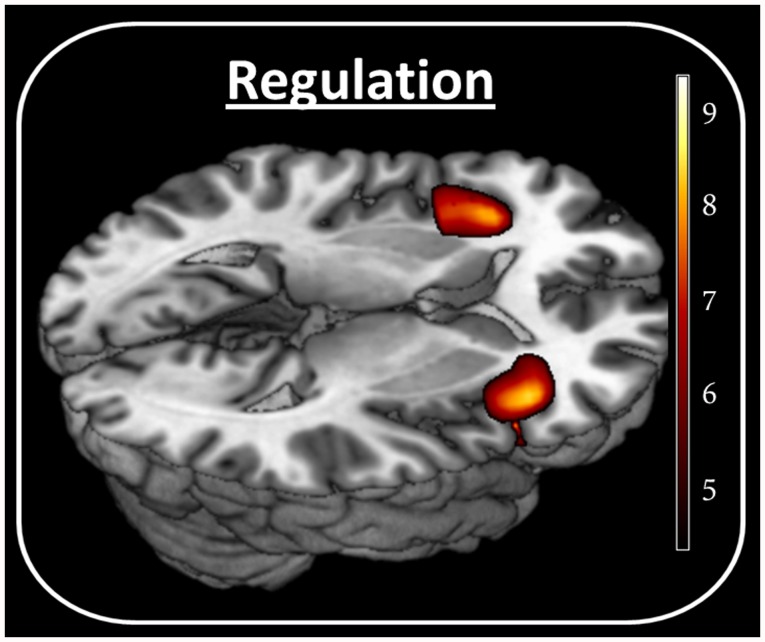
Regulation vs. relaxation. Activation in the SPM contrast ‘upregulation – relaxation’ in the AIC bilaterally for all subjects (right: −36 26 −2, T = 7.85; left: 39 20 −5, T = 7.65). Color bar shows T values.

**Figure 2:**
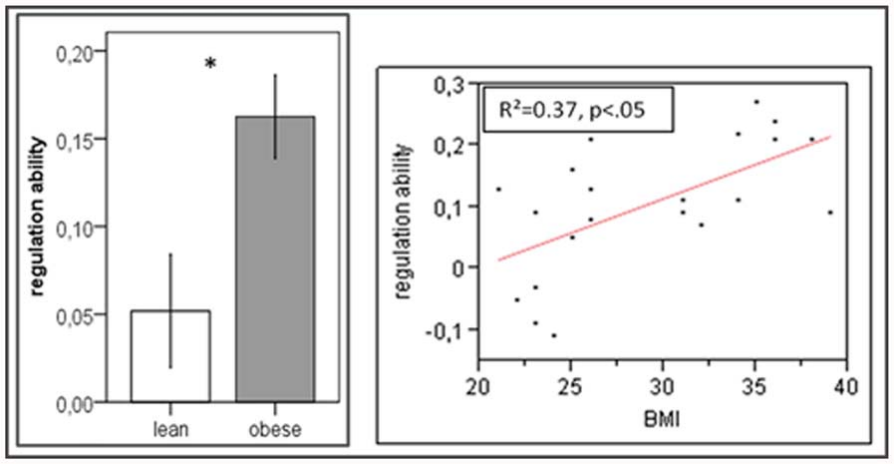
Regulation ability. Left: Bar graph of differences in the regulation ability between lean and obese participants. The bar-plots represent mean regulation ability ± SEM. Right: Scatterplot and regression line of the regulation ability and BMI.

A significant upregulation-effect was found in the AIC bilaterally in both groups (Figure 1). The RAI, however, revealed a significant group difference, indicating enhanced regulation ability in obese participants (*t*(19) = 2.791, *P* = 0.01) (Figure 2 left). Correlation analysis revealed a positive correlation between the BMI and the regulation ability (*R* ^2^ = 0.37, *P*<0.05) (Figure 2 right). Four lean participants were not able to regulate (RAI≤−0.03) and were therefore excluded from further connectivity analysis.

### Functional Connectivity

**Figure 3:**
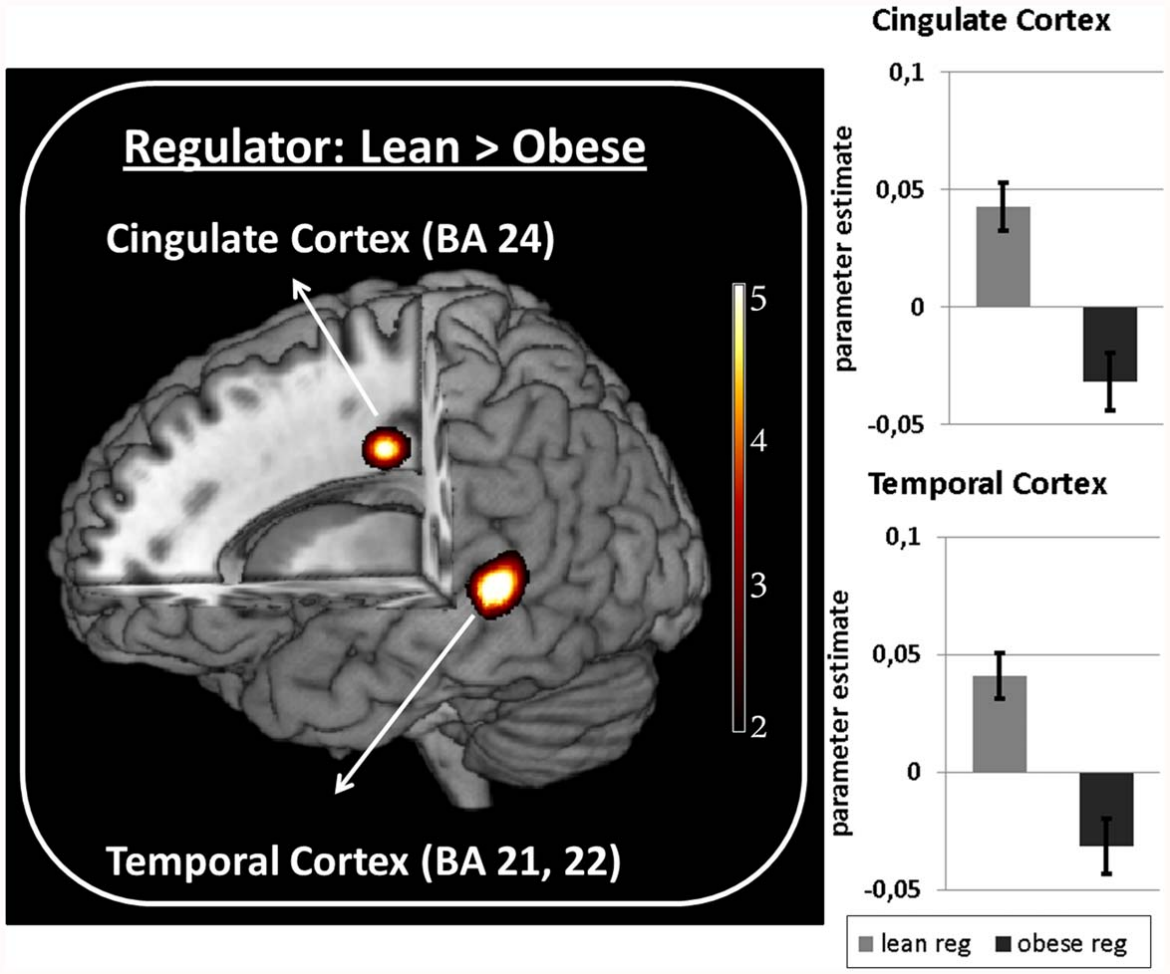
Connectivity differences in lean and obese. Left: Group differences in connectivity strength of the AIC with cingulate (−11 −17 36, T = 4.71) and temporal regions (−59 −35 0, T = 4.79) for lean regulators vs. obese regulators. Color bar shows T values. Right: Bar plots represent parameter estimates ± SEM.

Lean compared to obese regulators showed stronger connectivity to the medial cingulate cortex as well as to the medial temporal cortex during upregulation (Figure 3).

### Behavioral Data

The analysis of the behavioral data revealed no differences between lean and obese in the hunger rating or in the FCQ-S on both days. Furthermore, no significant differences were observed on the *restraint* and *experienced hunger* domain, however, higher scores in the *disinhibition* scale for obese participants in the TFEQ (t(19) = 2.67, *P*<0.05). Mood variables (anxiety, power of concentration, anger, sadness, happiness, nervousness, weakness) did not show significant differences between the groups pre and post the measurements (Table 2).

**Table 2:** Mood parameters.

	Lean				Obese				p-value
	day 1		day 2		day 1		day 2		
	pre	post	pre	post	pre	post	pre	post	
anxiety	9.6±4.5	10.6±7.6	1.8±1.5	0.6±0.6	8.5±3.2	3.2±1.1	2.6±1.1	1.3±0.6	n.s.
concentration	50.1±8.5	34.6±6.3	37.5±10.6	30.8±7.9	48.7±10.0	43.6±4.2	40.4±8.9	35.7±4.8	n.s.
anger	4.8±3.0	6.4±3.7	0.5±0.5	2.6±2.3	6.2±3.0	2.0±0.7	5.2±1.9	3.2±1.2	n.s.
sadness	9.5±5.2	4.1±2.3	3.5±1.6	3.0±1.9	12.8±5.6	1.6±0.7	5.7±3.4	3.8±1.3	n.s.
happiness	35.8±8.1	28.2±7.2	37.3±8.9	45.0±8.8	33.2±5.3	34.3±6.7	40.8±3.9	40.2±6.3	n.s.
nervousness	16.6±5.7	11.8±5.9	8.0±3.7	3.6±2.2	22.6±9.9	9.7±5.0	13.8±5.7	5.5±4.4	n.s.
weakness	16.1±7.2	19.4±5.6	6.0±4.8	12.7±6.2	17.3±7.1	23.4±8.2	7.9±3.7	13.8±6.0	n.s.

Mean ± SEM in mm of mood parameters pre and post fMRI measurements rated on a 100 mm visual analogue scale. P-values result from multivariate ANOVA.

n.s. = not significant (P>0.05).

## Discussion

In the present study, we investigated the ability to regulate the activity of AIC by using rtfMRI-BCI in lean and obese men. All obese participants were able to upregulate the AIC, while four of the lean participants were not successful in the self-regulation. In conjunction with previous findings showing a positive correlation between AIC activity and BMI [19] and the crucial role of the AIC in emotional stimuli processing [20], the enhanced AIC self-regulation may imply that obese men are more sensible to hedonic gustatory learning and can better shift their attention to emotional and body-related stimuli. Since the AIC is involved in emotional processing and hence showing affect-related response modulation, we asked the subjects to rate several mood parameters before and after the measurement. These ratings, however, did not show significant group differences and do, therefore, not contribute to the explanation of the BMI related differences. To get a first insight in the mechanisms behind these differences, we calculated in a further step the connectivity between the AIC and the rest of the brain on a preliminary base. The comparison of lean and obese regulators revealed stronger connectivity during upregulation of the AIC with the medial cingulate and medial temporal cortex in lean regulators. The connectivity between these regions represents the core-network of the brain, which is involved in task specific processes [21], cognitive states [22], and cognitive appraisal [23]. We, therefore, speculate that lean participants have to increase network connectivity to perform insular activation equally to the obese participants. This is in line with the hypothesis that obese people are more likely to activate their gustatory cortex after stimulation with food items [6]–[9]. The AIC responds to the salience of interoceptive stimuli, including motivational, emotional, cognitive or homeostatic states across tasks, through underlying autonomic processes [21]. Furthermore, resting state fMRI studies revealed that the temporal and the cingulate cortex are functionally connected to the AIC [24] and that the connectivity within the temporal network including the insular cortex is decreased in obese compared to lean participants [11]. This suggests a stable pattern of differences of functional connectivity between lean and obese both task-dependent and task-independent. However, we want to point out that the functional connectivity analysis in the present study was used in an exploratory manner and the results have to be interpreted cautiously based on the small number of subjects. So far, there are only few studies evaluating predictors for successful self-regulation performance. There are indications that the initial performance [25], motor imagery [26] as well as a high aptitude in BCI using [27] are best predictors for BCI performance. Thus, interoceptive-gustatory learning may also constitute a positive predictor for successful self-regulation of the AIC which is slightly positively correlated with obesity because of a tighter associative neural network of eating-related interoceptive functions in obese persons. The ability to intervene directly on the brain by voluntarily regulation of eating-related regions could be used to establish a tool to increase the control of such brain regions and affect eating-related behavior. However, this goal may require the regulation of the whole brain network representing gustatory and reward functions and their connections. In addition, further studies may use this approach to modulate regions involved in behavioral control and inhibition, e.g. frontal cortex. Therefore, the current study provides first evidence that BCI provides a new avenue to a better understanding of obesity and possible innovative therapeutic approaches.

This study is limited by the small number of participants especially after exclusion of participants who were not able to regulate. In addition, we chose the AIC as the region of interest; however, this is of course only one brain region involved in gustation and reward processing. Other gustatory regions (e.g. orbitofrontal cortex) or network related feedback might be better suited for regulation training in obese. Additionally, also down-regulation of such target regions can be implemented. The crucial further experiments in this direction have to establish eating behavior related changes by voluntary control of brain activity. This was a preliminary study to adapt fMRI-BCI training to obese people, but further research is needed to evaluate its therapeutic benefit.
